# Predicting the risk of invasion by broadleaf watermilfoil (*Myriophyllum heterophyllum*) in mainland Portugal

**DOI:** 10.1016/j.heliyon.2024.e34201

**Published:** 2024-07-05

**Authors:** Iúri Diogo, Neftalí Sillero, César Capinha

**Affiliations:** a Institute of Geography and Spatial Planning, University of Lisbon, Rua Branca Edmée Marques, 1600-276 Lisboa, Portugal; b CICGE – Research Centre on GeoSpatial Sciences, Faculty of Sciences, University of Porto, Alameda do Monte da Virgem, 4430-146 Vila Nova de Gaia, Portugal; c Centre of Geographical Studies, Institute of Geography and Spatial Planning, University of Lisbon, Rua Branca Edmée Marques, 1600-276 Lisboa, Portugal; d Associate Laboratory Terra, Portugal

**Keywords:** Aquatic invasions, Biodiversity modeling, Environmental suitability, Introduction risk, Species distribution modeling

## Abstract

Broadleaf water milfoil (*Myriophyllum heterophyllum*) is an emerging invasive alien plant in Europe, and thus a priority for European Union (EU)-level surveillance, monitoring, and eradication. This species is native to North America and threatens aquatic ecosystems by creating dense stands that can fill an entire water body, leading to high economic costs and the loss of native biodiversity. Although its presence in Portugal is not reported, the species has already been established in several European countries, including neighboring Spain. In this study, we assessed the risk of invasion by this species in mainland Portugal by jointly considering environmentally suitable areas and the risk of human-mediated introduction. Environmental suitability was estimated using MaxEnt, which relates the known species distribution to climate, topography, and soil variables. The model achieved a mean area under the curve value of 0.96 ± 0.008 and identified the mean temperature of the warmest quarter as the most relevant variable for explaining the species distribution (67.2 %). Predictions from the model indicated that the peaks of suitability values were distributed mainly in temperate climate regions along central and northern coastal areas in Portugal. The risk of introduction was estimated by mapping and calculating the spatial density of the aquarium stores. Jointly considering environmental suitability and risk of introduction, we observed that hotspots at risk of invasion are concentrated on the Portuguese central and northern coasts and in the two main metropolitan areas, Lisbon and Porto. Several risk hotspots fall within protected areas and sites designated under the EU Habitats Directive, comprising water bodies of high significance for maintaining local vegetation and fauna. Therefore, it is necessary to take measures to reduce the risk of invasion by this species, namely, surveillance and monitoring efforts confirming its absence in the national territory and preventing its future arrival.

## Highlights

• The risk of invasion by broadleaf water milfoil in mainland Portugal was assessed.
• Suitable areas for this aquatic plant are widespread, including in protected areas.
• Sites likely for the species introduction coincide spatially with suitable areas.

## 1 Introduction

Globalization of human travel and trading activities facilitates the spread of various plant species into regions outside their native ranges [[1], [2], [3]], increasing the number of invasive species and their impacts. Invasive alien species are the main causes of biodiversity loss [4] and have substantial negative implications for human health [5,6]. In addition, the total estimated cumulative cost of biological invasions in Europe alone was 140.20 billion US dollars between 1960 and 2020 [7].

The aquatic plant *Myriophyllum heterophyllum* is listed as an “invasive alien species of Union concern,” a subset of species considered a priority for European Union (EU)-level actions of surveillance, monitoring, and eradication owing to the observed or potential negative consequences on native biodiversity. This species is an evergreen submerged perennial aquatic plant called broadleaf water milfoil or variable-leaf water milfoil [[8], [9], [10]], and it is native to eastern North America and an alien species in New England [8,9,11,12] and Canada (New Brunswick, Prince Edward Island, Quebec, and Ontario) [13]. Moreover, its presence in Germany has been known since the 1940s, in Germany [12]. During the last decade, its presence has been reported in nine other countries, i.e., Austria, Belgium, France, Hungary, the Netherlands, Spain, Switzerland, the United Kingdom, and Croatia [6,9,14]. However, given the wide range of physical and chemical conditions that this species can tolerate [15], its range may encompass most of Europe [10,15], including Portugal.

Like other aquatic plants, variable-leaf water milfoil reproduces mainly through auto-fragmentation or allo-fragmentation and can be regenerated from fragments smaller than 1 cm [9,12,16], and its fragments have a slower than average desiccation rate, can efficiently preserve moisture, and remain viable for long periods [9,14,17]. This species was probably introduced to waterways in non-native American regions through wastewater disposal from aquarium stores, as it is popularly used in aquariums and aquatic gardening [12,18,19]. Other ways of anthropogenic dissemination include the importation of plants for scientific investigation and fragments transported in clothing, shoes, machinery (e.g., engines or trailers), boats, fishing, or sports/recreational aquatic equipment from waters colonized by the species [9,12,14,18]. Natural dispersal occurs when fragments become entangled in waterfowl or are created by disturbances caused by fish and their subsequent transport in watercourses, occasionally enhanced by flooding, wave action, or wind [9,12,18].

The species generally prefers lotic ecosystems, such as riparian systems, slow-flowing rivers, estuaries, canals, irrigation channels, lakes, ditches, reservoirs, and semi-aquatic systems, including swamps [6,9,20,21]. Moreover, it regularly inhabits low-elevation water bodies with pH levels of 6–7 and sediments consisting mainly of organic matter [10,14,20,22,23]. *Myriophyllum heterophyllum* does not perish during winter or summer and can tolerate low winter temperatures, water covered by ice, and high summer temperatures [9,12,21], and the most favorable temperature range for its growth is 18–25 °C [12]. Once introduced, *M. heterophyllum* can grow at explosive rates [18], which can be attributed to its evergreen growth and its capacity to use HCO_3_ as a carbon source, offering an advantage over seasonal native species and obligate CO₂ users [14,24].

Consequently, *M. heterophyllum* often creates dense mats that fill the entire surface and depth of water bodies [6,10], dominating other submerged macrophytes. This dominance reduces the availability of sunlight and oxygen in water bodies, potentially resulting in biodiversity loss [6,9,12,21]. Moreover, it alters predator-prey relationships [9], hampers water-based recreational activities such as swimming, diving, and fishing [16,22,25,26], and diminishes the aesthetic and monetary value of properties adjacent to affected lakes [18]. Furthermore, when *M. heterophyllum* invades drainage and irrigation systems, it can significantly impact water availability and flow, which may interfere with hydroelectric power production [9,26]. Despite these impacts, the potential distribution of the species in EU countries, especially Portugal, remains unknown, hindering the prioritization of prevention and surveillance actions at regional to local scales.

In this study, we assessed areas of mainland Portugal that were susceptible to the invasion of *M. heterophyllum*. This species has not yet been detected in the country; however, its naturalization in Spain [23] raises significant concerns, considering the presence of similar climatic conditions and because invasions in Spain often precede those occurring in mainland Portugal [2,27,28]. To perform this assessment, we first identified the environmental conditions suitable for the establishment of the species using MaxEnt and followed best modeling practices [29,30]. Next, we estimated the risk of the introduction of this species along the territory as a function of the density of aquarium stores, as these are the main centers from where this plant is disposed of, and then it enters the water bodies [12,18]. Finally, we combined the estimates of habitat suitability and the risk of introduction to highlight areas most susceptible to invasion in mainland Portugal.

## 2 Methods

### 2.1 Data collection

**Fig. 1:**
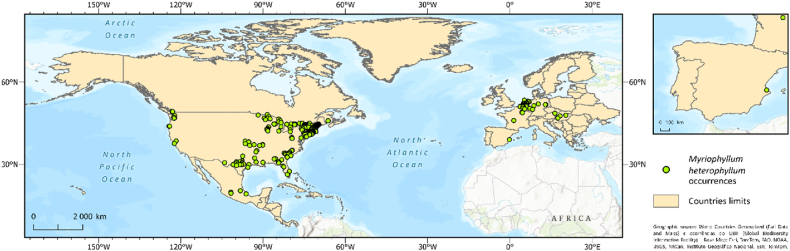
Occurrence records used for modeling the potential distribution of *Myriophyllum heterophyllum*.

To model the environmental suitability of the species, we collected occurrence records of its global distribution from the Global Biodiversity Information Facility (https://www.gbif.org/). We used records generated since 2000 onward in both native (America) and non-native ranges (Europe) (Fig. 1) to capture, as much as possible, the species’ environmental requirements [31]. Duplicate records, those without coordinates, or those with a spatial resolution of <10 m were removed. Additionally, to mitigate spatial bias, a single randomly selected record was retained within a 30 arc-s grid cell (i.e., the same resolution as that of the predictive variables [32]).

To assess areas at a high risk of introduction events, i.e., those under propagule pressure [33], we collected the geographical coordinates of shops registered as aquarium stores and “generic” pet stores. To confirm the sale of aquarium products for the latter category, we used available data from online sources (e.g., photographs of the social networks of the establishment or products for sale on the respective websites). Only the shops that confirmed to sell aquarium products were considered. We identified 329 stores in mainland Portugal.

Before their use for modeling, we calculated the pairwise Pearson correlations among the variables. Subsequently, we removed the highly correlated values until no correlation value higher than |0.7| occurred [38]. After this procedure, nine predictor variables (water_bodies, sediments, pH, altitude, T_Seas, T_Warmest, T_Coldest, P_ Annual, and P_Driest) were retained.

### 2.2 Predictor variables

Based on the analysis of the species’ known ecological preferences [9,10,14,20], we selected 17 predictive variables representing current climate, topography, and soil from multiple sources (complete list and the description of sources in Table 1) in Europe and North and Central America. We used the values of variables provided by the sources, except for the “amount of organic matter in the sediments,” where we joined six soil classes (Histosols, Chernozems, Kastanozems, Phaeozems, Umbrisols, and Podzols) to specify information regarding the presence or absence of organic soil. Therefore, we combined all cells with organic matter values equal to or exceeding 50 % from the six soil classes. We chose this percentage value because, upon overlapping all six soil classes, the entire study area would be covered by the layer; therefore, reclassifying only cells with values equal to or exceeding 50 %, we could identify only those cells exceeding 50 % concentration of organic matter. Next, to account for neighborhood effects, i.e., sediments transported in waterways, we calculated the density of these cells at each location considering a radius of 500 m. All variables were resampled to a 30 arc-s resolution (∼1 km) to match the resolution of the variables coarser than those with 30 arc-s resolution (e.g., climate; Table 1). All spatial procedures were performed using ArcGIS 10.8.2 software.

**Table 1:** Description and sources of predictive variables used.

Variable (code)	Description	Data source	Reference for the source	Spatial resolution (original)
Water_bodies	Water bodies	EarthEnv	Tuanmu e Jetz [34]	1 km
pH	Soil pH	SoilGrids	ISRIC; Batjes et al. [35]	250 m
Sediments	Sediments with organic matter			
Altitude	Altitude	EarthExplorer	USGS	7.5 arc-s
Slope	Slope	Derived from Altitude	–	7.5 arc-s
P_Annual	Average annual precipitation	CHELSA v. 2.1	Swiss Federal Institute for Forest, Snow, and Landscape Research WSL; Karger et al. [36,37]	30 arc-s (∼1 km^2^)
P_Driest	Average precipitation in the driest quarter			
P_Wettest	Average precipitation in the wettest quarter			
P_Mes_Dry	Precipitation in the driest month			
P_Mes_Wet	Precipitation in the wettest month			
P_Seas	Precipitation seasonality			
T_Annual	Average annual temperature			
T_Coldest	Average temperature in the coldest quarter			
T_Warmest	Average temperature in the warmest quarter			
T_Mes_Cold	Minimum daily average temperature in the coldest month			
T_Mes_Warm	Maximum daily average temperature in the hottest month			
T_Seas	Temperature seasonality			

### 2.3 MaxEnt — maximum entropy algorithm

Using MaxEnt 3.4.4, we modeled native and non-native ranges and then focused on mainland Portugal. MaxEnt is an open-source software written in Java that uses a machine-learning approach called maximum entropy to identify geographical regions with suitable environmental conditions for species [[39], [40], [41]]. MaxEnt uses species presence and background records [42] and often provides well performing predictions, even if based on limited, incomplete information, or both [41,[43], [44], [45], [46]]. MaxEnt is the most commonly used algorithm in the field of ecological niche models [47], including estimating environmental suitability for invasive species [48].

We converted all selected variables into ASCII format for use in the software. All variables were on a continuous scale, and we used the default parameters of the MaxEnt cloglog format as the output format; as a result, suitability values varied between 0 and 1, with a maximum of 10,000 background points.

We assessed the model performance using the area under the curve (AUC) of receiver operating characteristic plots [[46], [49]]. The AUC discriminates a species’ model from a random prediction, where the predictive ability of a model is considered “perfect” if the AUC value is 1.0 and “good” if the value is > 0.8 and a result of 0.5 does not discriminate better than when using randomly generated values [50,51]. We used 10 replicates to perform this evaluation, where the data were divided randomly into 10 groups (folds) of equal size, with one fold left out and the remaining nine used to fit the model. The AUC was calculated for data in the left-out fold. This procedure was repeated until all folds were used for evaluation. The final model corresponded to the mean and standard deviation models for evaluating these replicates.

We determined the contribution of each predictor variable to explain species distribution using the permutation method, which was performed in MaxEnt by shuffling the values of each variable across the training data and assessing the resulting drop in the AUC. Shuffled variables that resulted in a higher reduction in the AUC value were of greater importance than those resulting in a lower reduction in the AUC value.

### 2.4 Density of aquarium stores and combination with habitat suitability estimates

To calculate the spatial density of aquarium stores, we used the ArcGIS 10.8.2 “Kernel Density” tool using the planar method. After calculating the spatial density of stores, the resulting raster was linearly normalized to vary between 0 (lowest density) and 1.0 (highest density).

We combined both layers by multiplying the value for the spatial density of stores with the value of the environmental suitability model to obtain a “final” invasion risk estimate. The values of the resulting layers varied between 0 and 1.0. A value of 0 indicates locations where the risk of introduction or environmental suitability is the lowest. Conversely, a value of 1.0 denotes areas with the highest levels of environmental suitability and risk of introduction, i.e., high “invasion risk.”

To better identify areas of higher susceptibility to the potential invasion of the species and guide surveillance actions, we overlapped the limits of national protected areas and sites designated under the Habitats Directive of the Natura 2000 Network with predictions of invasion risk.

## 3 Results

**Fig. 2:**
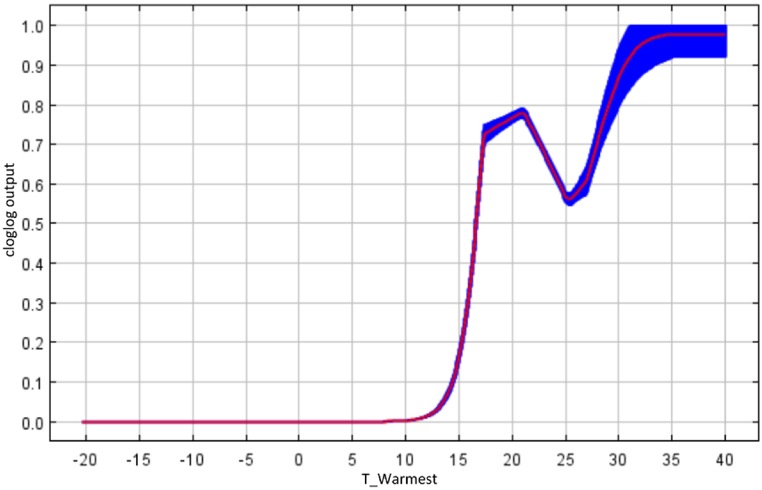
Response curve for temperature in the warmest quarter, i.e., the predictor variable with the highest relative importance in explaining the recorded distribution of *Myriophyllum heterophyllum*.

We obtained 524 occurrence records of *M. heterophyllum* in a known global range (Fig. 1). The habitat suitability model achieved extremely high predictive performance, with a mean AUC value of 0.96 ± 0.008. In this model, in the study region comprising Europe, North America, and Central America (see Supplementary Information), the variable temperature of the warmest quarter showed a disproportionally higher relative importance (67.2 %) than that of the remaining variables, with the remaining variables providing only minor to exceptionally minor contributions (Table 2). The response curve for the temperature of the warmest quarter (Fig. 2) shows that temperatures below ∼13 °C translate into a complete absence of suitability for the species, while peak suitability occurs at temperatures above ∼35 °C.

**Table 2:** Permutation importance of each variable.

Variable	Permutation Importance
Average temperature in the warmest quarter	67.2
Temperature seasonality	8.6
Average temperature in the coldest quarter	7.1
Average annual precipitation	6.3
Average precipitation in the driest quarter	4.2
Soil pH	2.8
Altitude	1.8
Water bodies	1.5
Sediments with organic matter	0.6

The predictions at the continental scale (Fig. S1) show that suitable environments in North America are predominantly observed along the eastern United States, extending from the Gulf of Mexico to southeastern Canada. A particular concentration of areas with high environmental suitability values exists along the coastal regions of the Carolinas, progressing toward the northeastern states and encompassing the regions of New York, Maine, New Brunswick, and Nova Scotia. Moreover, certain regions in the Netherlands and Belgium were identified as the most suitable areas in Europe, followed by hotspots in France, northern Spain, and northwestern Portugal. Generally, the southern regions of Europe and the Scandinavian countries are predicted to have lower levels of environmental suitability than that of the other regions of Europe.

**Fig. 3:**
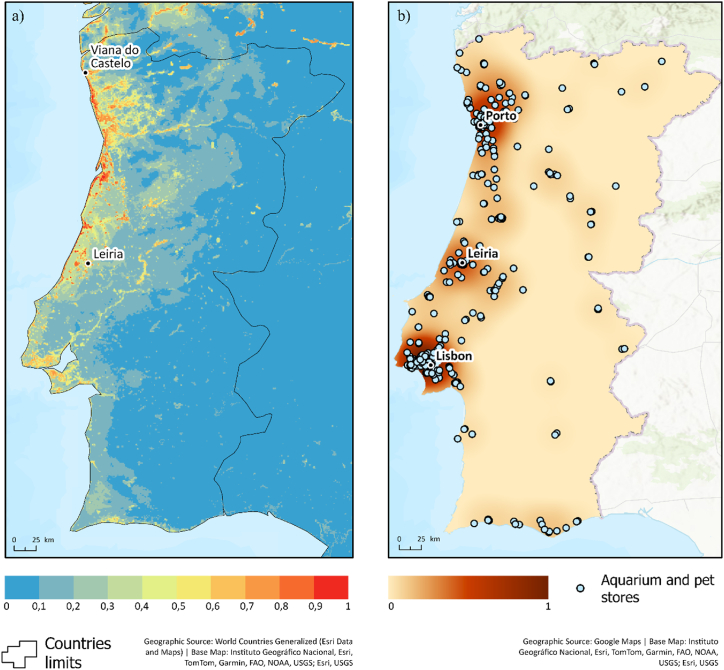
(A) Environmental suitability for *Myriophyllum heterophyllum* in mainland Portugal. **(B)** The density of aquarium stores in mainland Portugal.

In mainland Portugal, the highest environmental suitability values, peaking at 0.96, were observed along the northern coastal half, specifically in the region from Viana do Castelo to Leiria (Fig. 3A). A few areas in the southern coastal half exhibit moderate suitability values, ranging from 0.5 to 0.6, whereas, and inland regions are largely dominated by environmental conditions unsuitable to the introduction of invasive species. Regarding the distribution of aquarium stores, a high concentration of these stores was observed in a few coastal areas, particularly around the Lisbon Metropolitan Area (LMA), Porto Metropolitan Area (PMA), and Leiria (Fig. 3B).

**Fig. 4:**
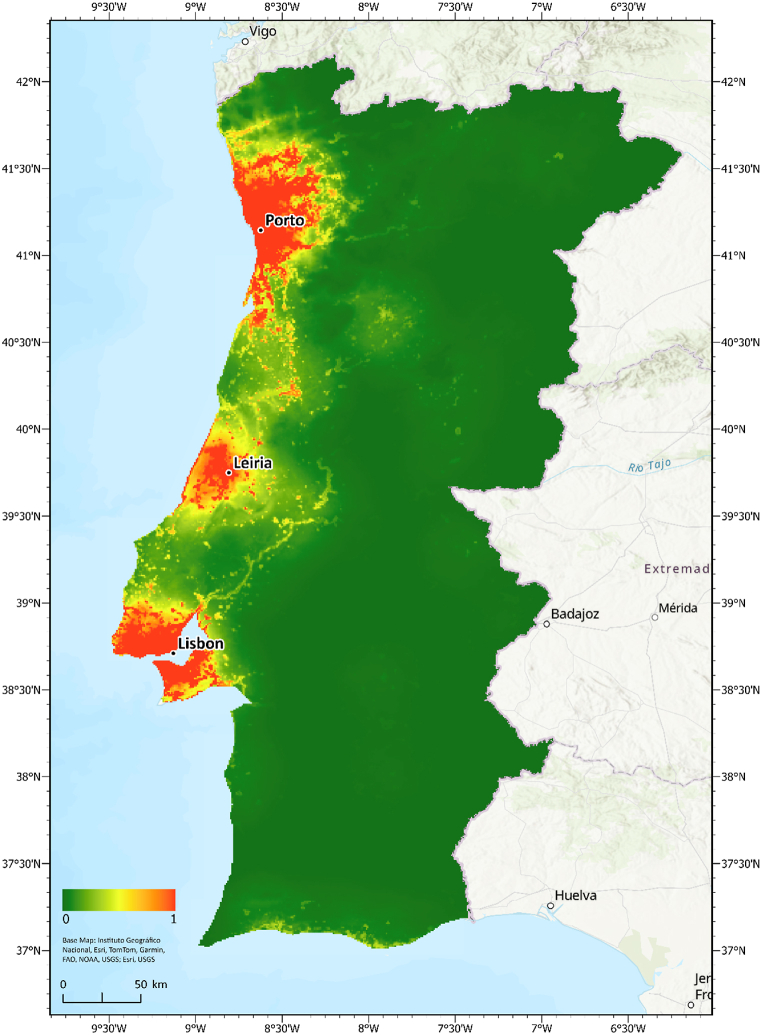
Hotspots with a high probability of occurrence of *Myriophyllum heterophyllum* in mainland Portugal. Estimated values jointly consider the degree of environmental suitability for the species and the likelihood of anthropogenic introductions based on the observed densities of aquarium stores.

The coastal areas around the LMA, PMA, and Leiria stood out as invasion hotspots, simultaneously hosting a high density of aquarium stores and having the highest habitat suitability values (Fig. 4). Areas in the interior of the country showed a low probability of invasion, owing to low store density and habitat suitability.

**Fig. 5:**
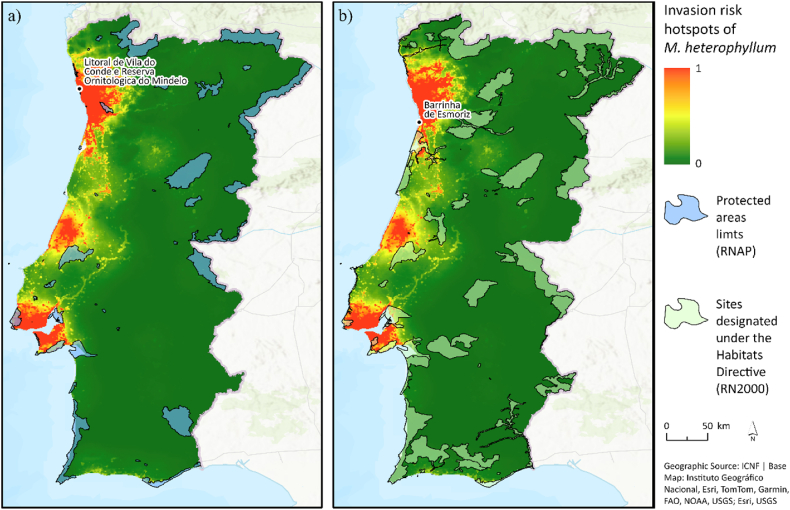
(A) Invasion risk hotspots of *Myriophyllum heterophyllum* overlapped by protected areas. **(B)** Sites designated under the European Union (EU) Habitats Directive (RN2000). Abbreviations: EU, European Union; RN2000, Natura 2000 Network of Habitats; and RNAP, National Network of Protected Areas.

The overlap between the environmental suitability and the risk of invasion with the national network of protected areas (Fig. 5A) and sites designated under the Habitats Directive (RN2000) (Fig. 5B) shows that many classified areas have suitable environmental conditions for the species, and a relevant number have a high risk of invasion (i.e., both high environmental suitability and probability of introduction).

## 4 Discussion

Our results indicate that the aquatic plant *M. heterophyllum* presents a high risk of invasion into the inland waters of the coastal areas of mainland Portugal. We observed incredibly high environmental suitability values (≥0.75) for this species, with its highest concentration occurring along the northwest coastal areas, from the district of Viana do Castelo to Leiria. When combined with an estimate of the probability of anthropogenic introductions, we observed prominence in the northwestern region of Portugal, specifically in the Lisbon, Porto, and Leiria regions. This pattern aligns with previous findings wherein northwest mainland Portugal was identified as the region with the highest diversity of invasive alien species in riparian zones [52]. Similarly, two areas in this region, Beira Litoral and Douro Litoral, were identified as having the highest rates of the introduction of alien plant species [53]. Thus, the high propensity for invasions by alien plants in this region further corroborates our findings regarding the high risk of invasion by *M. heterophyllum*.

A relevant number of protected areas showed a high risk of invasion, many of which, e.g., Litoral de Vila do Conde e Reserva Ornitológica do Mindelo Regional Protected Landscape and the Barrinha de Esmoriz site (see Fig. 5), comprise waterbodies of high importance for local vegetation and fauna and rare and endemic plant species, e.g., *Coincya johnstonii* and *Jasione lusitanica*. Therefore, the consequences of uncontrolled invasion by *M. heterophyllum* in the identified areas could be highly detrimental to local biodiversity. In addition, the colonization areas of non-native species recurrently coincide with the areas of agricultural expansion [54], and *M. heterophyllum* is a known threat to the farming sector, invading drainage and irrigation systems and reducing the availability and flow of water [9,26].

Based on the results of the MaxEnt model, climatic variables are the most relevant predictors of the potential distribution of the species, supporting the assertion of Rodríguez-Merino et al. [55] that the ability of alien plant species to invade new regions in Europe depends mainly on climatic conditions. The average temperature in the warmest quarter has the highest relative importance. Temperature directly affects reproduction, survival, growth, and dispersal in freshwater species and indirectly affects their interactions with other species in the habitat [55,56]. Mild to high temperatures during the warmest quarter of the year were associated with higher environmental suitability values for the plant, which is in agreement with previous knowledge indicating the most favorable temperature range for the growth of this species [9,12,21]. In contrast, we observed little relevance of precipitation-related predictors for the introduction of this species; however, despite this limitation, these predictors indicate water availability throughout the year and are linked to the distribution, transport, and establishment capacity of this species [9,12,18,55]. The reason for this result remains unascertained and may reflect the supracontinental extent of the model calibration area, with extensive regions characterized by wet climates and the wide availability of water bodies, likely masking the limiting effects of this variable. Despite this outcome, the Portuguese climate is expected to shift toward a relatively more severe semi-arid state owing to climate change [57], likely leading to a decrease in the number and size of lotic ecosystems, thereby reducing the extent of suitable habitats for *M. heterophyllum*.

Our work identified potentially suitable areas for the species, although further refinement may be possible by considering factors deemed relevant at a finer resolution for which comprehensive spatial data are currently unavailable. For example, the successful invasion and survival rates of *M. heterophyllum* have been linked to higher lake orders than to lower lake orders. Higher lake orders are characterized by elevated alkalinity, conductivity, and flushing rates resulting from substantial water inputs from ion-rich groundwater sources [20,58]. Furthermore, the physiography of the receiving habitat plays a crucial role in the establishment of plant fragments because barriers such as stones or vegetation can restrict their drift distance [58]. Therefore, given the availability of data, future research assessing the potential distribution of the species could greatly benefit from incorporating these factors. In addition, considering the role of temperature as a key determinant of the potential distribution of this species, future assessments should be conducted by analyzing the implications of representative climate change scenarios in the future. However, this assessment extends beyond the scope of this study and concentrates on identifying the regions of priority for immediate invasion prevention efforts.

Additionally, it is crucial to conduct field surveys in the identified risk hotspots based on our results to confirm the absence of this species. If confirmed, several measures could be implemented to reduce the risk of its spread, such as inspecting boating or agricultural equipment used in invaded waters to prevent transport to novel areas, similar to practices in countries such as Canada [12]. Additionally, for small established populations, eradication methods such as careful hand pulling could prove effective [8,15]. Preventive strategies could include intensifying inspection efforts related to the possession and importation of the species and educational campaigns aimed at the aquarium community to raise awareness of *M. heterophyllum*.

## 5 Conclusions

Considering the existence of invasive locations in Spain, it may only be a matter of time before *M. heterophyllum* reaches mainland Portugal. Our results indicate the presence of environmental conditions in Portugal that are suitable for the establishment of this species and, worryingly, overlap with high-risk areas for human-mediated introduction, primarily in the northwestern coastal regions of the country. Despite being listed among the prohibited species, information about this species is scarce in Portugal, which reflects a lack of awareness among the general population. The results of our study could inform decision-making concerning preventive measures against the invasion of this species.

### Ethics declaration

Review and approval by an ethics committee or informed consent was not required for this study because it did not involve human subjects or laboratory experiments.

## Data availability statement

Species occurrence data are available from the GBIF (DOI: https://doi.org/10.15468/dl.qqkdnk). Data on the predictor variables are publicly available from the sources indicated in the text.

## Funding

César Capinha received funding support from the Portuguese 10.13039/501100019370Foundation for Science and Technology (10.13039/501100001871FCT) awarded to the CEG/IGOT Research Unit (UIDB/00295/2020 and UIDP/00295/2020). Neftalí Sillero was supported by the CEEC2017 contract (CEECIND/02213/2017) from 10.13039/501100001871FCT.

## CRediT authorship contribution statement

**Iúri Diogo:** Writing – original draft, Visualization, Software, Project administration, Methodology, Investigation, Formal analysis, Data curation, Conceptualization. **Neftalí Sillero:** Writing – review & editing, Methodology. **César Capinha:** Writing – review & editing, Validation, Supervision, Software, Project administration, Methodology, Formal analysis, Conceptualization.

## Declaration of competing interest

The authors declare that they have no known competing financial interests or personal relationships that could have appeared to influence the work reported in this paper.

## References

[1] Seebens H., Blackburn T.M., Dyer E.E., Genovesi P., Hulme P.E., Jeschke J.M., Pagad S., Pyšek P., Winter M., Arianoutsou M., Bacher S., Blasius B., Brundu G., Capinha C., Celesti-Grapow L., Dawson W., Dullinger S., Fuentes N., Jäger H., Kartesz J., Kenis M., Kreft H., Kuhn I., Lenzner B., Liebhold A., Mosena A., Moser D., Nishino M., Pearman D., Pergl J., Rabitsch W., Rojas-Sandoval J., Roques A., Rorke S., Rossinelli S., Roy H.E., Scalera R., Schindler S., Stajerová K., Tokarska-Guzik B., van Kleunen M., Walker K., Weigelt P., Yamanaka T., Essl F. (2017). No saturation in the accumulation of alien species worldwide. Nat. Commun..

[2] Capinha C., Essl F., Porto M., Seebens H. (2023). The worldwide networks of spread of recorded alien species. Proc. Natl. Acad. Sci. USA.

[3] Ascensão F., Capinha C., Borda-de-Água L., Barrientos R., Beja P., Pereira H.M. (2017). Railway Ecology.

[4] Bellard C., Cassey P., Blackburn T.M. (2016). Alien species as a driver of recent extinctions. Biol. Lett..

[5] Meyerson L.A., Mooney H.A. (2007). Invasives and globalization LA Meyerson and HA Mooney 200. Front. Ecol. Environ..

[6] European Comission (2017). http://ec.europa.eu/environment/nature/pdf/IAS_brochure_species.pdf.

[7] Haubrock P.J., Turbelin A.J., Cuthbert R.N., Novoa A., Taylor N.G., Angulo E., Ballesteros-Mejia L., Bodey T.W., Capinha C., Diagne C., Essl F., Golivets M., Kirichenko N., Kourantidou M., Leroy B., Renault D., Verbrugge L., Courchamp F. (2021).

[8] Les D.H., Mehrhoff L.J. (1999). Introduction of nonindigenous aquatic vascular plants in southern New England: a historical perspective. Biol. Invasions.

[9] Anderson L., Fried G., Gunasekera L., Hussner A., Newman J.R., Starfinger U., Stiers I., van Valkenburg J., Tanner R. (2015). Pest risk analysis for *Myriophyllum heterophyllum*. http://nora.nerc.ac.uk/id/eprint/511890/.

[10] Jasprica N., Lasić A., Hafner D., Cetinić A.B. (2017). European invasion in progress: *Myriophyllum heterophyllum* michx. (Haloragaceae) in Croatia. Nat. Croat..

[11] Moody M.L., Les D.H. (2002). Evidence of hybridity in invasive watermilfoil (Myriophyllum) populations. Proc. Natl. Acad. Sci. USA.

[12] Lafontaine R.-M., Beudels-Jamar R.C., Delsinne T.D., Robert H. (2013).

[13] Canadensys (2021). http://www.canadensys.net.

[14] Gross E.M., Groffier H., Pestelard C., Hussner A. (2020). Ecology and environmental impact of *Myriophyllum heterophyllum*, an aggressive invader in European waterways. Diversity.

[15] EPPO (2016). Myriophyllum heterophyllum Michaux. Bulletin OEPP/EPPO Bulletin.

[16] Bailey J.E. (2007).

[17] Barnes M.A., Jerde C.L., Keller D., Chadderton W.L., Howeth J.G., Lodge D.M. (2013). Viability of aquatic plant fragments following desiccation. Invasive Plant Sci. Manag..

[18] Halstead J.M., Michaud J., Hallas-Burt S., Gibbs J.P. (2003). Hedonic analysis of effects of a nonnative invader (*Myriophyllum heterophyllum*) on New Hampshire (USA) lakefront properties. Environ. Manag..

[19] Thum R.A., Zuellig M.P., Johnson R.L., Moody M.L., Vossbrinck C. (2011). Molecular markers reconstruct the invasion history of variable leaf watermilfoil (*Myriophyllum heterophyllum*) and distinguish it from closely related species. Biol. Invasions.

[20] Thum R.A., Lennon J.T. (2009). Comparative ecological niche models predict the invasive spread of variable-leaf milfoil (*Myriophyllum heterophyllum*) and its potential impact on closely related native species. Biol. Invasions.

[21] GISD, Global Invasive Species Database (2021). http://www.iucngisd.org/gisd/species.php?sc=1700.

[22] Kimball K.D., Baker A.L. (1981). Littoral Waters.

[23] Prieto J.A.C., Liendo D., García-Magro D., Biurrun I. (2017). en los pozos de La Arboleda (Trapagaran, Bizkaia).

[24] Dülger E., Hussner A. (2017). Differences in the growth and physiological response of eight Myriophyllum species to carbon dioxide depletion. Aquat. Bot..

[25] Onion A.M., Gross E. (2004). Herbivore Resistance in Invasive and Native Myriophyllum Spicatum and Myriophyllum Heterophyllum.

[26] Tsiamis K., Gervasini E., Deriu I., D'Amico F., Katsanevakis S., Cardoso A.C. (2019). Baseline distribution of species listed in the 1st update of Invasive Alien Species of Union concern.

[27] Oficialdegui F.J., Zamora-Marín J.M., Guareschi S., Anastácio P.M., García-Murillo P., Ribeiro F., Oliva-Paterna F.J. (2023). A horizon scan exercise for aquatic invasive alien species in Iberian inland waters. Sci. Total Environ..

[28] Anastácio P.M., Banha F., Capinha C., Bernardo J.M., Costa A.M., Teixeira A., Bruxelas S. (2015). Indicators of movement and space use for two co-occurring invasive crayfish species. Ecol. Indicat..

[29] Sillero N., Arenas-Castro S., Enriquez‐Urzelai U., Vale C.G., Sousa-Guedes D., Martínez-Freiría F., Real R., Barbosa A.M. (2021). Want to model a species niche? A step-by-step guideline on correlative ecological niche modelling. Ecol. Model..

[30] Sillero N., Barbosa A.M. (2021). Common mistakes in ecological niche models. Int. J. Geogr. Inf. Sci..

[31] Beaumont L.J., Gallagher R.v., Thuiller W., Downey P.O., Leishman M.R., Hughes L. (2009). Different climatic envelopes among invasive populations may lead to underestimations of current and future biological invasions. Divers. Distrib..

[32] Zhang Z., Capinha C., Usio N., Weterings R., Liu X., Li Y., Yokota M. (2020). Impacts of climate change on the global potential distribution of two notorious invasive crayfishes. Freshw. Biol..

[33] Leung B., Mandrak N.E. (2007). The risk of establishment of aquatic invasive species: joining invasibility and propagule pressure. Proc. Biol. Sci..

[34] Tuanmu M.N., Jetz W. (2014). A global 1-km consensus land-cover product for biodiversity and ecosystem modelling. Global Ecol. Biogeogr..

[35] Batjes N.H., Ribeiro E., van Oostrum A., Leenaars J., Hengl T., Mendes de Jesus J. (2017). WoSIS: providing standardised soil profile data for the world. Earth Syst. Sci. Data.

[36] Karger D.N., Conrad O., Böhner J., Kawohl T., Kreft H., Soria-Auza R.W., Zimmermann N.E., Linder P., Kessler M. (2017). Climatologies at high resolution for the Earth land surface areas. Sci. Data.

[37] Karger D.N., Conrad O., Böhner J., Kawohl T., Kreft H., Soria-Auza R.W., Zimmermann N.E., Linder H.P., Kessler M. (2018). Data from: climatologies at high resolution for the earth's land surface areas. EnviDat.

[38] Dormann C.F., Elith J., Bacher S., Buchmann C., Carl G., Carré G., Marquéz J.R.G., Gruber B., Lafourcade B., Leitão P.J., Münkemüller T., Mcclean C., Osborne P.E., Reineking B., Schröder B., Skidmore A.K., Zurell D., Lautenbach S. (2013). Collinearity: a review of methods to deal with it and a simulation study evaluating their performance. Ecography.

[39] Phillips S.J., Anderson R.P., Schapire R.E. (2006). Maximum entropy modeling of species geographic distributions. Ecol. Model..

[40] Pearson R.G. (2010). Species' distribution modeling for conservation educators and practitioners. Lessons in Conservation.

[41] Phillips S.J., Anderson R.P., Dudík M., Schapire R.E., Blair M.E. (2017). Opening the black box: an open-source release of Maxent. Ecography.

[42] Guillera-Arroita Gurutzeta, Lahoz-Monfort José J., Elith Jane (2014). Maxent Is Not a Presence-Absence Method: A Comment on Thibaud et Al. Methods Ecol. Evol..

[43] Hernandez P.A., Graham C.H., Master L.L., Albert D.L. (2006). The effect of sample size and species characteristics on performance of different species distribution modeling methods. Ecography.

[44] Akasaka M., Osawa T., Ikegami M. (2015). The role of roads and urban area in occurrence of an ornamental invasive weed: a case of *Rudbeckia laciniata* L. Urban Ecosyst..

[45] Xu Z. (2015). Potential distribution of invasive alien species in the upper Ili river basin: determination and mechanism of bioclimatic variables under climate change. Environ. Earth Sci..

[46] Byeon D.H., Jung S., Lee W.H. (2018). Review of CLIMEX and MaxEnt for studying species distribution in South Korea. J. Asia Pac. Bus..

[47] Abdelaal M., Fois M., Fenu G., Bacchetta G. (2019). Using MaxEnt modeling to predict the potential distribution of the endemic plant Rosa arabica Crép. in Egypt. Ecol. Inf..

[48] Miller J. (2010). Species distribution modeling. Geography Compass.

[49] Liu C., White M., Newell G. (2009). Measuring the accuracy of species distribution models: a review. 18th World IMACS Congress and MODSIM09 International Congress on Modelling and Simulation: Interfacing Modelling and Simulation with Mathematical and Computational Sciences, Proceedings.

[50] Phillips S.J., Schapire R.E. (2004). Proceedings of the 21st International Conference on Machine Learning.

[51] Zhang Z., Capinha C., Weterings R., McLay C.L., Xi D., Lü H., Yu L. (2019). Ensemble forecasting of the global potential distribution of the invasive Chinese mitten crab, *Eriocheir sinensis*. Hydrobiologia.

[52] Pabst R., Dias F.S., Borda-de-Água L., Rodríguez-González P.M., Capinha C. (2022). Assessing and predicting the distribution of riparian invasive plants in continental Portugal. Frontiers in Ecology and Evolution.

[53] Marchante E., Marchante H. (2016). World Sustainability Series.

[54] Rodríguez-Merino A., Fernández-Zamudio R., García-Murillo P. (2017). An invasion risk map for non-native aquatic macrophytes of the Iberian Peninsula. Anales Del Jardin Botanico de Madrid.

[55] Rodríguez-Merino A., García-Murillo P., Cirujano S., Fernández-Zamudio R. (2018). Predicting the risk of aquatic plant invasions in Europe: how climatic factors and anthropogenic activity influence potential species distributions. J. Nat. Conserv..

[56] Gallardo B., Aldridge D.C. (2013). The «dirty dozen»: socio-economic factors amplify the invasion potential of 12 high-risk aquatic invasive species in Great Britain and Ireland. J. Appl. Ecol..

[57] Soares P.M., Lima D.C. (2022). Water scarcity down to earth surface in a Mediterranean climate: the extreme future of soil moisture in Portugal. J. Hydrol..

[58] Heidbüchel P., Sachs M., Stanik N., Hussner A. (2019). Species-specific fragmentation rate and colonization potential partly explain the successful spread of aquatic plants in lowland streams. Hydrobiologia.

