# Supplementary material for: Predicting the risk of invasion by broadleaf watermilfoil (Myriophyllum heterophyllum) in mainland Portugal
Source: Heliyon. 2024 Jul 5;10(13):e34201. doi: 10.1016/j.heliyon.2024.e34201 (PMC11283033; doi:10.1016/j.heliyon.2024.e34201)
Supplement: Multimedia component 1 [file mmc1.docx]

**Heliyon**

**Supplementary Information for**

Predicting the risk of invasion by broadleaf watermilfoil (*Myriophyllum heterophyllum*) in mainland Portugal

Author list: Iúri Diogo, Neftalí Sillero, César Capinha

***Corresponding author:** Iúri Diogo | iuridiogo@campus.ul.pt

**This PDF file includes:**

Figure S1

**Supplementary Figures**


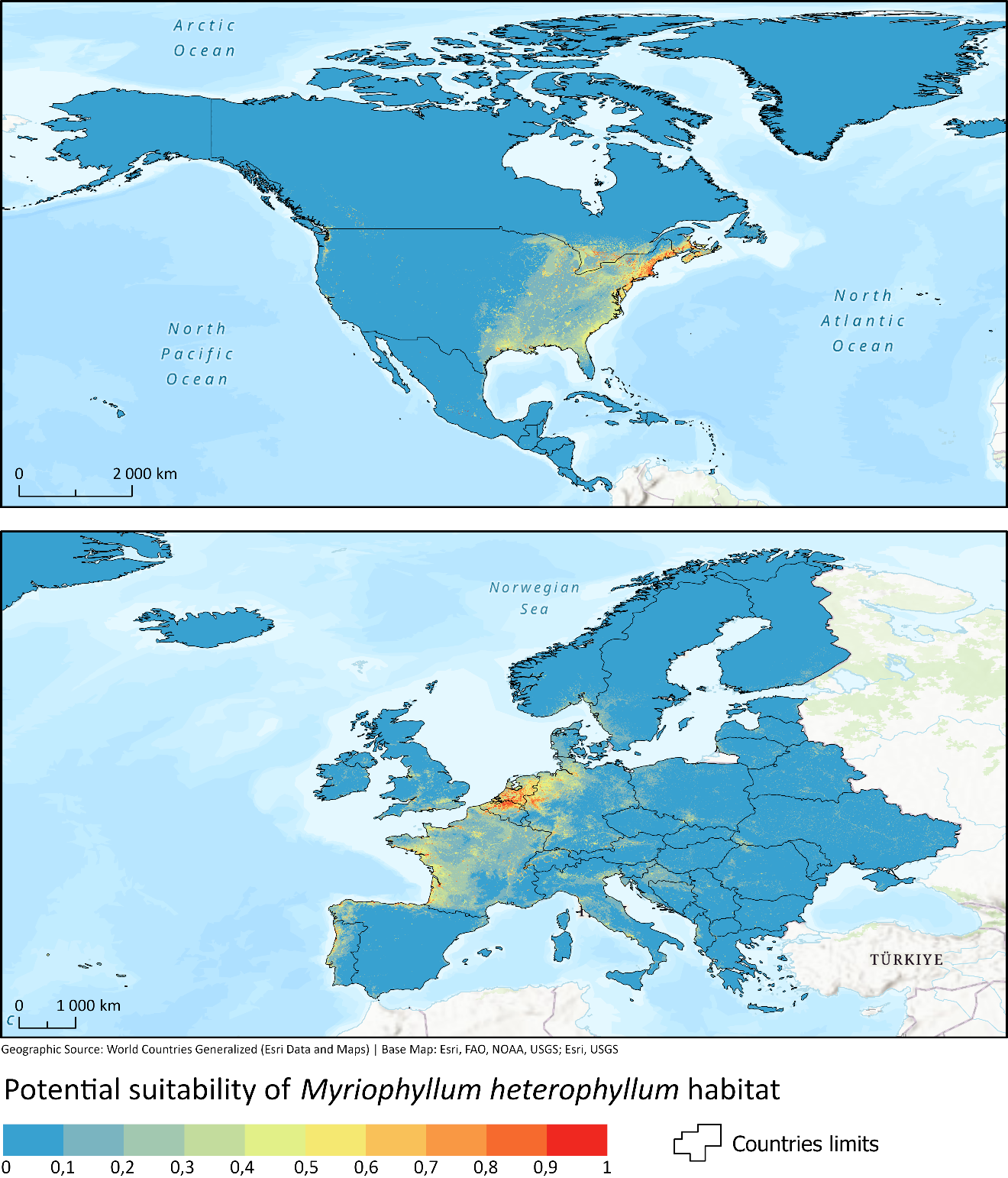


**Figure S1.** Environmental suitability for *Myriophyllum heterophyllum*
